# Olfactory ensheathing cells travel their natural nasal pathway to deliver therapeutics to brain tumors

**DOI:** 10.18632/oncotarget.27043

**Published:** 2019-07-09

**Authors:** Litia A. Carvalho, Bakhos A. Tannous

**Affiliations:** ^1^ Experimental Therapeutics and Molecular Imaging Laboratory, Department of Neurology, Neuro-Oncology Division, Massachusetts General Hospital, Boston, MA, USA; Neuroscience Program, Harvard Medical School, Boston, MA, USA

**Keywords:** olfactory ensheathing cells, glioma, gene therapy

Gliomas are the most prevalent primary tumors occurring in the central nervous system (CNS), with an incidence of more than 9,000 patients per year in the United States alone. Glioblastoma (GBM) is the most frequent subtype of gliomas in adults, presenting with very aggressive and invasive nature, conferred by high cellular proliferation and migration, resulting in recurrence. Despite aggressive therapy that includes surgical resection combined with radiation and chemotherapy (temozolomide, TMZ), the five-year survival rate of patients with GBM remains less than 10% after initial diagnosis [1]. Treatment failure and tumor recurrence are not only related to GBM high invasive nature, but also to tumor cellular heterogeneity, with a subpopulation of glioma stem-like cells (GSCs; also known as glioma initiating cells), notorious for their resistance to conventional treatment. Further, the blood-brain barrier (BBB) prevents efficient drug delivery to gliomas, complicating even further treatment of this aggressive disease.

**Figure 1:**
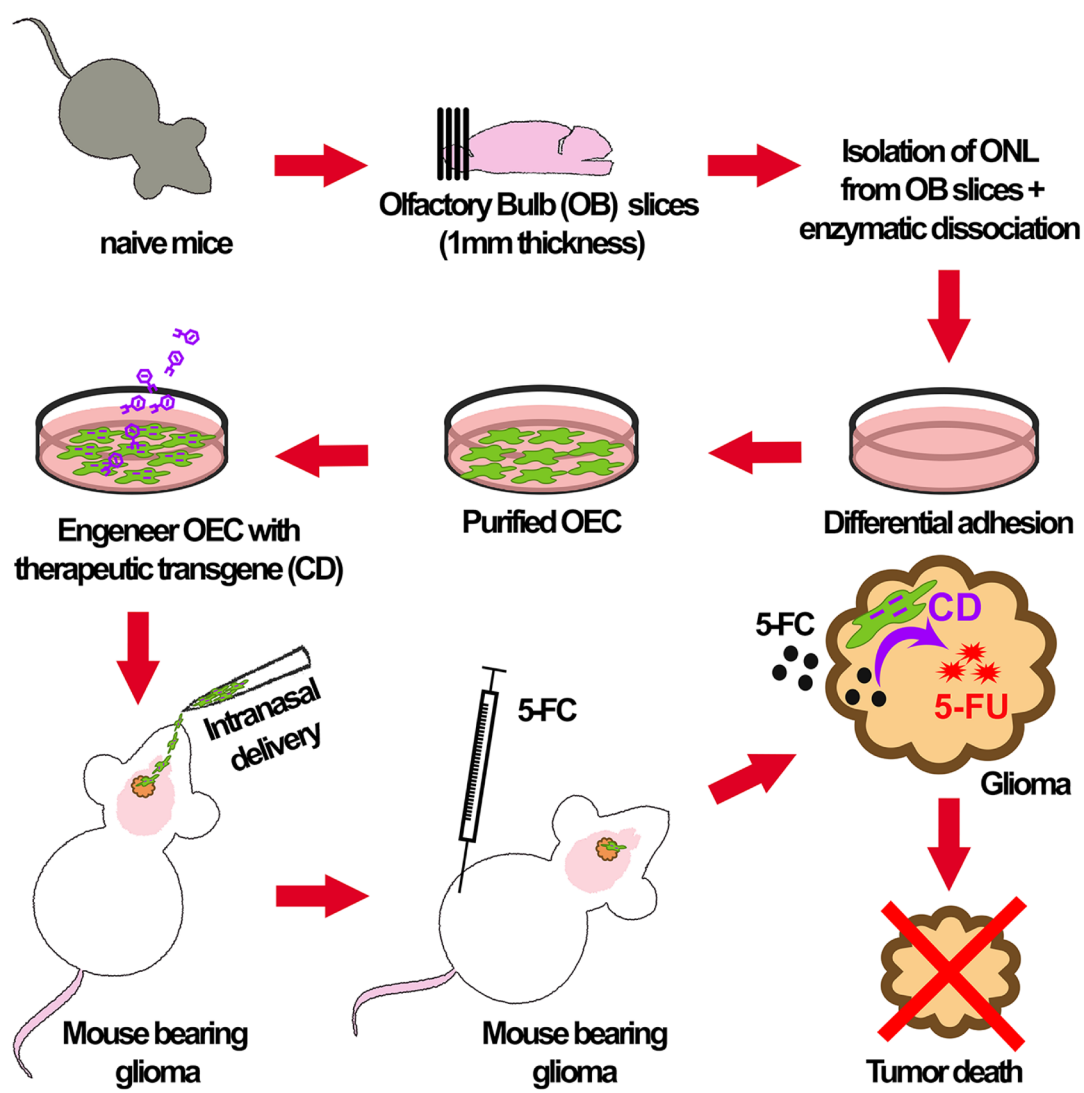
Schematic representation of olfactory ensheathing cells isolation and modification for glioma gene therapy. The olfactory bulb (OB) is dissected from naive mice, and the olfactory nerve layer (ONL) is then removed and dissociated. Fibroblasts, astrocytes and oligodendrocytes are first removed by adhesion to a culture dish, while the floating OEC cells are plated onto laminin-coated plate in growth media. OEC cultures are expanded, and once reaching confluency, OECs are engineered by a lentivirus vector to express a therapeutic transgene such as cytosine deaminase (CD). These modified OECs are then administered by nasal drops into mice-bearing brain tumors, to travel their natural route to the central nervous system, targeting brain tumors and infiltrative glioma stem cells. Mice are then injected with the pro-drug 5-FC which is converted to an active metabolite 5-FU at the tumor cells, through OEC-expressing CD, leading to tumor regression.

For the past 20 years, engineered stem cells, such as neural stem cells (NSCs) and mesenchymal stem cells (MSCs), have been used as a vehicle to carry and deliver anticancer agents to a variety of brain tumors, including gliomas [2]. These cell-based vehicles are centered on the natural tropism of stem cells to tumor inflammatory cues, targeting and delivering the therapeutic payload only to tumor cells while sparing healthy tissue, minimizing collateral effects. Further, these stem cells can bypass the BBB upon systemic injection. Although NSC and MSC are largely studied for the treatment of gliomas, other cell types that share similar properties but more advantageous could expand the repertoire of these therapies. The terminally differentiated olfactory ensheathing cell (OEC) is a unique glia that ensheaths the axons of the olfactory neuronal sensors in the olfactory mucosa, guiding and promoting their extension until their final target into the CNS, the olfactory bulb. OEC plays a major role in the continuous replenishment of the olfactory receptors, a process that occurs throughout all mammalian lifespan, by isolating the new generated axons from the non-regenerative permissive environment [3]. OEC displays several features of astrocytes and Schwann cells, conferring the ability to interact with both cell types directly, thus, being the only glial cell present in both the peripheral nervous system (PNS) and CNS [4]. Owing to these as well as immuno-modulatory and phagocytic properties, OECs therapeutic potential was evaluated in the clinic for different neurological pathologies and regenerative medicine including chronic spinal cord injury, stroke, and amyotrophic lateral sclerosis [5, 6], but were never studied in the context of cancer. In a recent report published in the journal of the National Cancer Institute, we showed for the first time OEC tropism to brain tumors and their potential use as a “Trojan horse” to deliver therapeutic transgene [7]. OEC reach the brain and selectively target the primary tumor site as well as infiltrative/invasive GSCs upon intranasal administration, their natural route to CNS (Figure 1). Further OEC could be modified by a lentivirus vector to express and deliver a therapeutic transgene (cytosine deaminase) to brain tumors, and is able to convert the systemically administered pro-drug 5-fluorocytosine (5-FC) into the active chemotherapeutic 5-fluorouracil (5-FU), at the tumor site, leading to a bystander killing of GBM cells and an increase in mice survival (Figure 1).

OEC is the main phagocytic cell in the olfactory epithelium (and not macrophages), and are responsible for cellular debris clearance as a result of neuronal replenishment during physiological turnover of neurons, or after olfactory nerve lesion [8]. As such, OEC is very receptive to the local inflammatory environment required for wound healing. Several studies have shown that OEC is very responsive to (and can secrete) many proinflammatory cytokines at the lesion site, including interleukin 6 (IL-6) and tumor necrosis factor-alpha (TNF-alpha), enhancing their migration and proliferation rate, and playing a role in modulating the local inflammatory environment for wound healing [9, 10]. Indeed, we showed that these cytokines also play a key role in OEC tropism to gliomas [7].

The use of OECs for GBM gene/cell therapy has several advantages over traditional stem cells: (1) OEC is a fully differentiated cell (and not stem cell) that can be easily obtained from the olfactory epithelium and/or bulb, a simple procedure typically performed for patients with spinal cord injury or pituitary tumor resection, allowing autologous transplantation, avoiding graft-versus-host disease; (2) OEC migration can be stimulated by cytokines associated with tumor growth, such as TNF-α and IL-6, or even by low-doses of curcumin, a known antitumor agent; (3) the intranasal pathway is a non-invasive alternative to deliver therapeutics to the brain, and is the natural route for OEC during olfactory neuron turnover; (4) No toxic or tumorigenic potential with OECs transplantation have been reported to date; Finally, given their immunomodulatory and phagocytic properties, OEC have the potential to be used not only as a vehicle to deliver therapeutic genes, but as adjuvant therapy opening new possibilities for glioma treatment that can be extended to other cancer types.

Given OECs therapeutic potential for neurological pathologies and regenerative medicine, many studies are underway to optimize their collection, culturing and expansion techniques. For instance, a current clinical trial (NCT02870426) aiming at collecting OEC from brain dead or living donors undergoing elective neurosurgery and using them for patients with spinal cord injury (not autologous). As these studies evolve, they will offer a tremendous benefit in the application of OEC to GBM therapy. In all clinical applications thus far, OEC are injected directly into the area of pathology, while the proposed intranasal delivery might require higher number of cells, direct injection of OECs at the tumor site should also be explored in future studies.
